# Water Treatment Practices and Misperceived Social Norms among Women Living with Young Children in Rural Uganda

**DOI:** 10.4269/ajtmh.23-0723

**Published:** 2024-07-09

**Authors:** Jessica M. Perkins, Bernard Kakuhikire, Charles Baguma, Meredith Meadows, Raphael Abayateye, Justin D. Rasmussen, Emily N. Satinsky, Patrick Gumisiriza, Justus Kananura, Elizabeth B. Namara, David R. Bangsberg, Alexander C. Tsai

**Affiliations:** ^1^ Department of Human and Organizational Development, Peabody College, Vanderbilt University, Nashville, Tennessee;; ^2^ Vanderbilt University Medical Center, Nashville, Tennessee;; ^3^ Mbarara University of Science and Technology, Mbarara, Uganda;; ^4^ Department of Psychology, Duke University, Durham, North Carolina;; ^5^ Center for Global Health, Massachusetts General Hospital, Boston, Massachusetts;; ^6^ Department of Psychology, University of Southern California, Los Angeles, California;; ^7^ VinUniversity, Hanoi, Vietnam;; ^8^ Harvard Medical School, Boston, Massachusetts;; ^9^ Mongan Institute, Massachusetts General Hospital, Boston, Massachusetts

## Abstract

Access to water safe for consumption is critical for health and well-being, yet substantial structural barriers often necessitate household action to make water safer. Social norms about water treatment practices are understudied as a driver of personal water treatment practice. This study assesses reported and perceived water treatment practices among women in a rural, water insecure setting. We used cross-sectional data from a population-based study of women living with children under 5 years old across eight villages in southwest Uganda. Participants reported their typical household water treatment practices and what they perceived to be the common practices among most other women with young children in their own village. Modified multivariable Poisson regression models estimated the association between individual behavior and perceptions. Of 274 participants (78% response rate), 221 (81%) reported boiling water and 228 (83%) reported taking at least one action to make water safer. However, 135 (49%) misperceived most women with young children in their village not to boil their water, and 119 (43%) misperceived most to take no action. Participants who misperceived these norms were less likely to practice safe water treatment (e.g., for boiling water, adjusted relative risk = 0.80; 95% CI 0.69–0.92, *P* = 0.002), adjusting for other factors. Future research should assess whether making actual descriptive norms about local water treatment practices visible and salient (e.g., with messages such as “most women in this village boil their drinking water”) corrects misperceived norms and increases safe water treatment practices by some and supports consistent safe practices by others.

## INTRODUCTION

Approximately two billion people worldwide retrieve their water from a feces-contaminated water source.^1^ More than 800,000 people die each year from diarrhea as a result of unclean drinking water, and 220 million receive treatment each year for having parasitic diseases from consuming unclean drinking water.^1^ Water insecurity is associated with many additional adverse outcomes, including malnutrition, infant health, personal injury, emotional distress, violence against women, and weakened community cohesion.^2^^–^^10^ In eastern and southern Africa, poor access to clean water, sanitation, and hygiene causes more than half of overall disease burden.^11^ Systematically providing clean water to rural populations in eastern and southern Africa would cost an estimated $5 billion.^12^ In the absence of structural solutions, individual households bear the burden of procuring clean water for household consumption, typically through point-of-use household water treatment practices and safe water storage.^13^^–^^15^

The United Nations Sustainable Development Goal 6 on water and sanitation includes ensuring universal and equitable access to safe and affordable drinking water for all by 2030.^16^ In eastern and southern Africa, only 30% of people are able to use safely managed drinking water services.^16^ Educational and economic barriers often prevent uptake of safe water treatment practices.^13^^,^^17^^,^^18^ However, many strategies to decrease barriers and improve water treatment practices have not sufficiently increased take-up of safe practices.^19^ Implementing novel strategies aiming to improve health and development outcomes in water insecure contexts requires identifying other factors that influence safe water treatment practices. Misperceptions about most others’ water treatment practices (i.e., misperceived norms) as drivers of personal water treatment practices are an understudied opportunity for intervention.

### Conceptual framework.

Social norms are conceived of as the shared unwritten rules that govern and constrain individual and social behavior.^20^^,^^21^ Decades of research on social norms indicate that humans follow what others do.^22^^–^^24^ Fear of receiving sanctions, being seen as part of the out-group, being isolated, wanting to be part of the in-group, and feeling identified with local groups can elicit conformity and motivate people to feel and behave how they think others do. Thus, perceived injunctive norms (i.e., beliefs about what most other support) and perceived descriptive norms (i.e., beliefs about what most others do) often influence behavior.^25^^,^^26^ Individuals form these perceptions about specific groups of people based on exposure to salient information, that is, visual or auditory cues in the environment.^27^

However, people often misperceive what it is that most others support and what most others do when asked about specific reference groups. Studies have found that people tend to incorrectly believe few people engage in health behaviors when in fact most do, and that many people engage in risk behaviors when in fact most do not.^28^^–^^32^ A lack of cues about health-promotive behavior, a focus on some (extreme) cues over other (e.g., less attention grabbing) cues, being in a group or network with outlier behavior, and various psychological attribution and conversation processes can lead to norm perception bias.^27^^,^^33^^–^^39^ These misperceptions matter because people act in line with their perceptions regardless of perception accuracy. A large body of work on perceived norms has shown evidence of both pervasive misperceived norms and strong links between misperceived norms and personal behavior across several fields and topics.^40^

People who misperceive norms are either in a state of false consensus^41^ or in a state of pluralistic ignorance.^42^ False consensus refers to when individuals incorrectly think that most others are like them (i.e., the state of thinking that most others do what I do or support what I support when in fact they do not). Pluralistic ignorance refers to when individuals incorrectly think that most others differ from them (i.e., the state of thinking that most others do not do what I do or support what I support when in fact they do). Both of these states are malleable as perceptions are malleable. Addressing misperceived norms among people in either state is important.

The social norms approach to behavior change harnesses these phenomena.^43^^,^^44^ The aim is to heighten the visibility and salience of actual local norms to change misperceived norms. In turn, people previously in a state of false consensus may change their behavior, and people previously in a state of pluralistic ignorance may change their outlook about their own behavior and become more vocal and visible about it and supportive of others to engage in the normative behavior. They may also be more likely to maintain their behavior and not succumb to effects of misperceived norms. A large body of research (including studies assessing causal effects) has shown that making actual health norms in relevant social groups more visible and salient can affect perceived norms and, in turn, lead to changes in individual and collective behavior.^45^^–^^58^

A review assessing sociopsychological determinants of safe water consumption practices across 14 studies identified perceived social norms as a critical factor driving household water treatment practices.^59^ Some additional recent studies also found that individuals’ perceptions about what most others do were associated with uptake of household water treatment among women caregivers in rural Nepal,^60^ among households in Indonesia,^61^ and among primary caregivers in Chad.^62^ No studies have assessed the type and extent of misperceived norms about water treatment practices in eastern and southern Africa nor how misperceptions may influence behavior. However, studies from this region on other health-related topics suggest that many individuals underestimate the prevalence of health behaviors and overestimate the prevalence of health risk behaviors, and that individuals’ perceptions of what most others do in their local environment influence their own behaviors.^63^^–^^69^

### The current study.

In rural Uganda, many households lack access to clean water and experience mild to severe water insecurity.^4^^,^^70^ This study used a unique whole-population study design targeting women with young children across eight villages in rural Uganda to assess the role of misperceived norms about water treatment practices on water treatment behavior in this context. Specifically, the design permitted direct comparison of perceptions about most other women’s water treatment practices with aggregated reports about personal practices and reports from neighbors’ about their own practices. This comparison thus identified misperceived norms about water treatment practices. Associations between misperceptions and individuals’ actual practices were then estimated while adjusting for exposure to neighbors’ water treatment practices, household water source, and sociodemographic characteristics. Findings can inform the design of novel strategies to promote household uptake of safe water treatment practices are in rural eastern and southern Africa contexts.^71^^,^^72^

## MATERIALS AND METHODS

### Study setting.

From 2016 through 2018, the study team conducted a population-based parent study on health and well-being. Specifically, this study targeted all resident adults across eight rural villages in Rwampara District, southwestern Uganda. The study setting was ∼260 km southwest of Kampala, the largest city in Uganda, and ∼20 km from the closest commercial hub, Mbarara City, which had a projected total population of 212,100 in 2018.^73^ The area contained eight small villages, which when grouped together are referred to as a parish. A parish is a local governance subunit. In rural areas in Uganda, parishes often contain several villages.

Almost all households within the study context have access to a stove and use firewood as the primary resource of fuel for cooking. Most households engaged in an agriculture-based economy, and a little more than half of households had a small solar power system providing enough electricity to charge small items like a mobile phone. Most households were experiencing daily household food and water insecurity.^4^^,^^70^^,^^74^^,^^75^ Approximately half of households with young children fetched water every day. Almost no households had their own rain-water harvesting tank, but water for daily needs was available from several private and public water sources distributed throughout the parish. They varied in quality from “improved” source (i.e., sheltered and protected from run-off contamination) to “unimproved” source (i.e., unprotected from contamination).^70^ Most of these sources were not safely managed drinking water services. Many were not free from fecal and priority chemical contamination, and many households did not have access points on their premises.^76^ Some water sources were reviewed on an ad hoc basis by local water committees for quality and operation.

### Study population.

Women in this setting are typically responsible for collecting and storing water, household activities that require water (e.g., cooking and cleaning), and caring for the sick (e.g., when a young child gets diarrhea). Although the parent study collected data on many topics, information about water source, quality, and treatment practices were collected only from women with at least one child under 5 years old in their home (hereafter referred to as women with young children). Because this was a population-based study, all households within the parish were targeted for inclusion in this study if they had at least one woman who met this criterion. For households with more than one eligible woman, the only data included for this study were from the oldest eligible woman in the household with a young child. Thus, the current study targeted one woman at least 18 years of age per household who was living with at least one child under 5 years. This study was purposefully set up to represent all households with young children in this setting. This unique design permits direct comparison of perceived local norms with the local prevalence of behaviors within these villages.

The study context was similar to where most people in rural Uganda live. Specifically, 75% of people in Uganda (and most people in many other eastern and southern African countries) live in outlying rural areas where the local economy features agricultural and small-scale trading and enterprise, access to electricity and to piped clean water is rare, and household food and water insecurity are common.^4^^,^^70^^,^^75^^,^^77^ Likewise, the sociodemographic characteristics of the study population are also similar to national sociodemographic characteristics. For example, most adults are married, most adults have never enrolled in or never completed secondary education, the adult population contains a large portion of persons who are 18–30 years old, and, among the minor population, a large portion who are under 5 years old.^77^^–^^79^

### Study procedures.

Community sensitization meetings open to all adults in the study setting were held before data collection to introduce the parent study to the targeted study population and respond to any questions.^80^ Additionally, a census was conducted in the targeted study setting to record all households within the eight villages and their permanent resident adults. At the time of the census, geographic location information was also collected for each household. Over the course of the data collection period, a team of research assistants who spoke the local language (Runyankore) approached eligible individuals, typically at their homes, and invited them to participate in the study. Eligible individuals who wished to participate provided written informed consent after learning about the study. A thumbprint plus a witness signature also indicated consent to participate. Research assistants then conducted one-on-one survey-based interviews typically at participants’ homes in a private location. Research assistants used a computer-assisted, survey-based interview tool to collect and record data. Survey questions had been through an iterative translation and piloting process between English and Runyankore to verify fidelity. Interviews lasted approximately 1 hour.

### Ethical approval.

Ethical approval was granted by the Partners Human Research Committee at Massachusetts General Hospital, the Research Ethics Committee at Mbarara University of Science and Technology, and the Vanderbilt Human Research Protections Program. We also received clearance from the Uganda National Council of Science and Technology and the Research Secretariat in the Office of the President of the Republic of Uganda.

### Measures.

#### Household water treatment practices.

To determine household water treatment practices, we asked each participant to report which action(s) they typically take to make their household drinking water safer. Specifically, we asked them if they 1) add bleach/chlorine to the water, 2) strain the water through a cloth, 3) boil the water, 4) use solar disinfection, 5) let the water stand and settle, 6) use another method to treat the water, or 7) take no action. Participants could report a yes or no response to each action except for the last option. Participants who reported taking no action could not report taking other actions.

#### Perceived norms about water treatment practices.

To assess perceptions about the local norm for water treatment practices, we asked each participant to indicate which of the same actions described above (e.g., add bleach/chlorine, strain through a cloth, etc.) that they thought most other women with young children in their own villages typically do to make their household drinking water safer. Participants could indicate a yes or no response about each action being typically done by most other women with young children in their own villages. Alternatively, participants could say most other women with young children take no action.

We chose “most women living with young children in your village” as the specific “local” reference network for eliciting individuals’ perceptions about water treatment norms for several reasons. First, gendered patterning of household labor in this setting suggests that women may identify more with other women regarding water treatment practices and may also feel like they have a better idea of what most local women do. Second, pilot testing indicated that study participants identified as belonging to a village, so the village was an easily understood social network. Moreover, village population sizes were small (ranging from 130 to 248 adults), and most people were socially connected within this community.^80^^,^^81^

#### Household water context.

Participants reported the main water source for their household and whether they thought that their household primarily used that source because it had acceptable water quality. Classification of sources according to Sustainable Development Goal standards was not available. However, household water insecurity was assessed using the Household Water Insecurity Access Scale (HWIAS), which consists of eight questions related to restricted water use (e.g., not drinking enough because water was not available or was too difficult to collect, worries about water access, and consuming/using water perceived to be unsafe or dirty).^82^ The HWIAS had been validated for use among Runyankore-speaking populations in Uganda.^70^ Composite HWIAS scores may range from 0 to 24 and were used to categorize household-level water access insecurity: water secure, mildly water insecure, moderately water insecure, and severely water insecure.

#### Neighbors’ household water treatment practices.

First, distances between participant households were computed using the geocoded location of each household. Relevant neighbors for a participant were defined as other participants in this study (i.e., women living with young children) who resided less than 0.2 km away from an individual participant based on haversine distance (i.e., the distance between two points on a spherical rather than flat surface). We used a 0.2-km threshold based on neighbor classification by a study of peer influence independently conducted in a similar context within the same rural area in Uganda.^83^ Using this definition of “neighbor,” we calculated for each participant the total number of neighboring households that had women living with young children. Then, we linked the data about neighbors’ reports of their typical household water treatment practices to each index participant and created a trichotomous variable: not having any neighbors; having one or more neighbor(s) who reported that they did not treat their household drinking water; and having one or more neighbors, all of whom reported that they treated their household drinking water. We included this variable as an additional explanatory factor because the behavior of proximal others reported by those proximal others could be associated with both personal water treatment practices and perceptions about the local norm for water treatment practice. This variable could be a confounder. Including it allows assessment of the association between perceptions about the local norm and personal water treatment practice independent from the influence of neighbors’ behavior.

## STATISTICAL ANALYSES

We first summarized household water treatment practices by participant characteristics, household water contexts, and neighbors’ behavior. The most common water treatment practice among a majority of women with children in each village was identified and hereafter referred to as the local norm for water treatment in each village. We then calculated and summarized misperceptions about local norms by participant characteristics, household water contexts, and neighbors’ behavior. This population-based study was designed so that study participants were included as both participants and as part of the social reference groups. With this design, the study team could directly compare local norms (based on aggregated reports of water treatment practices at the village-level) with perceived norms (participants’ perceptions about typical local water treatment practices among women with young children in their villages). This comparison identifies misperceived norms, i.e., when individuals’ perceptions differ from local norms. Prior research has used this method to identify misperceived norms about other topics.^63^^,^^64^^,^^67^

Finally, we fitted a multivariable modified Poisson regression model with village-level cluster-correlated robust estimates of variance where engaging in the most common safe water treatment practice was the binary outcome (e.g., boil versus do not boil) and misperception of the local norm was the primary explanatory variable. With a binary dependent variable, the exponentiated regression coefficients from the modified Poisson regression model have been shown to yield estimated incidence rate ratios that can be interpreted straightforwardly as relative risk ratios.^84^ The model adjusted for age, primary school completion, marital status, household asset wealth quintile,^85^ household water context factors (main source type, perceived water quality, and water insecurity), neighbors’ water treatment practice, number of neighbors, and whether the participant had experienced diarrhea in the past 7 days. We also conducted two sensitivity analyses. Specifically, we fit another model where “taking at least one action versus none” was the outcome. The misperception and neighbor variables were also changed accordingly. Additionally, we fit the original model and included a variable representing how firewood was obtained as primary source of fuel (from own land, from others’ land, purchased, or firewood was not main source of fuel). (This variable was also collected during the parent study.)

## RESULTS

The parent study had 775 households of which 350 had women living with young children. From this targeted population, 275 women with young children were interviewed and 274 provided data for the main outcome and explanatory variable (78% response rate). The number of participants per village ranged from 24 to 45 participants (with one participant per household). The number of close neighbors who were study participants (i.e., women living with young children) ranged from 0 to 16 people (median, 5; interquartile range, 3–8). Most participants had completed primary school (156 [57%]) and were married (234 [85%]). The average age was 36 years (SD = 9). Almost all participants (269 [98%]) reported using firewood as their primary source of fuel with 227 (84%), collecting it from their own land or others’ land.

Participants reported a range of household drinking-water sources: gravity flow scheme tap (88 [32%]), other kind of tap (47 [17%]), spring (68 [25%]), well (57 [21%]), and other source (14 [5%]). Approximately one-third of participants (91 [33%]) reported that the primary reason they used that source as their households’ main source of water was because of its acceptable water quality. Additionally, 83 (30%) reported that they had consumed water in the past 30 days that they thought might not be safe for their health. More than half of participants experienced household water insecurity, with 44 [17%]) experiencing severe water insecurity. One (<1%) participant declined to answer one or more of the questions describing their household’s water access insecurity. Few participants (14 [5%]) reported having diarrhea in the past 14 days.

Among 274 participants, 46 (16.8%) reported that they take no action treat their household drinking water to make it safer. However, 221 (80.7%) reported boiling the household drinking water. This was their only action. Five (1.8%) participants reported also only using one method, which excluded boiling water. Two (0.7%) participants reported using more than one method. The prevalence of boiling water ranged from 69% to 89% across villages, and most participants within each social stratum and household water context reported boiling their water (Table 1). Hereafter we refer to boiling water as the local norm.

**Table 1:** Household water treatment practices among women with children <5 years old across eight villages in Rwampara District, southwest Uganda (*N* = 274)

Variables	Participants		Boils Household Water to Make Safer for Consumption	
	*n*	%	*n*	%
Total	274	100	221	81
Age, Years				
18–29	66	24	52	79
30–39	121	44	98	81
40–49	70	26	56	80
≥50	15	6	13	87
Education	66	24	52	79
None/Some Primary Education	118	43	83	70
Completed Primary Education or More	156	57	138	88
Marital Status				
Not Married/Cohabiting	40	15	34	85
Married/Cohabiting as if Married	234	85	187	80
Household Asset Wealth				
1st (Quintile Poorest)	73	27	50	68
2nd Quintile	67	24	52	78
3rd Quintile	58	21	49	84
4th Quintile	43	16	37	86
5th (Quintile Least Poor)	33	12	33	100
Main Source for Household Drinking Water				
Gravity Flow Scheme	88	32	67	76
Other Kind of Tap	47	17	38	81
Well	57	21	52	91
Spring	68	25	51	75
Other	14	5	13	93
Acceptable Quality of Water is Primary Reason for Water Source				
No	151	55	124	82
Yes	91	33	72	79
Unknown	32	12	25	78
Household Water Insecurity				
Secure	120	47	94	78
Mild	25	10	22	88
Moderate	66	26	50	76
Severe	44	17	36	82
Neighbors’ Reports of Own Household Water Treatment Practices				
All Neighbors Take At Least One Action to Make Water Safer	119	43	106	89
Has One or More Neighbors Who Take No Action to Make Water Safer	142	52	105	74
Has No Close Neighbors	13	5	10	77
Diarrhea in Past 2 Weeks				
No	260	95	208	80
Yes	14	5	13	93

Despite this clear local norm, many participants did not believe that safe water treatment practice was common. Among 53 participants who did not boil their water, 40 (75%) were in a state of false consensus. They incorrectly thought that most others do not boil their household drinking water and they do not boil water themselves. Among 221 participants who boil their water, 95 (43%) were in a state of pluralistic ignorance. They incorrectly thought that most others do not boil their household drinking water but do boil water themselves. Overall, 16 participants (5.8%) incorrectly believed that letting water settle was the local norm among women with young children in their villages, 119 (43%) participants incorrectly believed that most women with young children in their villages do nothing to make their household drinking water safer, and 135 (49%) participants incorrectly believed that most women with young children in their villages do not boil their household drinking water to make it safer. These misperceptions were pervasive across all social strata and household water contexts (Table 2).

**Table 2:** Misperceptions about local household drinking water treatment practices among women with children <5 years old across eight villages in Rwampara District, southwest Uganda (*N* = 274)

Variables	Incorrectly Believed that Most Women with Young Children in Own Village Take No Action to Make Household Drinking Water Safer		Incorrectly Believed that Most Women with Young Children in Own Village do not Boil Household Drinking Water	
	*n*	%	*n*	%
Total	119	43	139	51
Age, Years				
18–29	34	52	40	61
30–39	48	40	56	46
40–49	32	46	34	49
≥50	5	33	5	33
Education				
None/Some Primary Education	55	47	59	50
Completed Primary Education or More	64	41	76	49
Marital Status				
Not Married/Cohabiting	12	30	15	38
Married/Cohabiting as if Married	107	46	120	51
Household Asset Wealth				
1st Quintile Poorest	36	49	41	56
2nd Quintile	30	45	31	46
3rd Quintile	20	34	29	50
4th Quintile	21	49	22	51
5th Quintile Least Poor	12	36	12	36
Main Source for Household Drinking Water				
Gravity Flow Scheme	54	61	60	68
Other Kind of Tap	17	36	19	40
Well	21	37	24	42
Spring	25	37	30	44
Other	2	14	2	14
Acceptable Quality of Water is Primary Reason for Water Source				
No	61	40	69	46
Yes	48	53	55	60
Unknown	10	31	11	34
Household Water Insecurity				
Secure	57	48	58	48
Mild	10	40	12	48
Moderate	29	44	34	52
Severe	22	50	24	55
Neighbors’ Reports of Own Household Water Treatment Practices				
All Neighbors Take At Least One Action to Make Drinking Water Safer	44	37	50	42
Has One or More Neighbors Who Take No Action to Make Drinking Water Safer	70	49	79	56
Has No Close Neighbors	5	38	6	46
Diarrhea in Past 2 Weeks				
No	116	45	131	50
Yes	3	21	4	29

Among the 135 participants who misperceived boiling water as not being the local norm, 95 (70%) reported that they boil their household drinking water. In contrast among the 139 participants who perceived that boiling water was the local norm, 126 (91%) reported that they boil their household drinking water (chi-square = 18.04; *P* <0.001). Similarly, among the 119 participants who misperceived no action as the local norm, 86 (72%) reported that they took at least one action to treat their household drinking water to make it safer for consumption. In contrast, among the 155 participants who perceived that taking at least one action to treat household water was the local norm, 142 (92%) reported that they took at least one action to treat their household drinking water (χ^2^ = 18.03; *P* <0.001).

Women’s water treatment practices remained associated with their misperceptions of the local norm for water treatment practices when adjusting for household water context, neighbors’ water treatment practices, and sociodemographic factors. Participants who misperceived the local norm about boiling water were less likely themselves to practice this safe water treatment method (adjusted relative risk = 0.80; 95% CI: 0.69–0.92, *P* = 0.002) compared with participants who believed most others boil their household drinking water to make it safer. The estimated association between misperceiving taking no action as the local norm and personal report of taking at least one action to make the household drinking water safer was similar. Not completing primary education was also associated with not boiling water and not taking any action (Table 3). Including whether firewood was primary source of fuel and if so where firewood was obtained did not change the pattern or strength of the estimated associations.

**Table 3:** Multivariable modified Poisson regression models estimating associations between reported household water treatment practice and misperceiving the local norm about safe water treatment practice among women living with young children across eight villages in Rwampara District, southwest Uganda (*N* = 253)

Variables	Household Boils Water to Make Safer for Consumption		
	aRR	95% CI	*P*-Value
Perception of Local Norm on Household Drinking Water Treatment Practices			
Misperceived Most Women with Young Children in Own Village to not Boil Drinking Water	0.80	0.69–0.92	0.002
Believed Most Women with Young Children in Own Village Boil Drinking Water	Ref	–	–
Age (in Years)	1.00	0.99–1.01	0.908
Married/Cohabiting as if Married (vs. Not Married)	0.90	0.79–1.02	0.107
Did not Complete Primary Education (vs. Completed Primary Education)	0.83	0.74–0.93	0.001
Household Asset Wealth			
1st Quintile (Poorest)	0.79	0.62–1.01	0.061
2nd Quintile	0.88	0.67–1.17	0.391
3rd Quintile	0.97	0.79–1.18	0.734
4th Quintile	Ref	–	–
5th Quintile (Least Poor)	1.06	0.86–1.30	0.584
Main Source of Household Drinking Water			
Gravity Flow Scheme	Ref	–	–
Other Kind of Tap	0.96	0.80–1.14	0.616
Well	1.07	0.95–1.21	0.249
Spring	0.90	0.70–1.17	0.432
Other	1.03	0.84–1.26	0.769
Acceptable Quality of Water is Primary Reason for Water Source			
No	Ref	–	–
Yes	1.02	0.96–1.09	0.466
Unknown	0.94	0.85–1.05	0.266
Household Water Insecurity			
Secure	Ref	–	–
Mild	1.11	0.95–1.30	0.195
Moderate	0.97	0.86–1.09	0.574
Severe	1.10	0.91–1.33	0.309
Neighbors’ Reports of Own Household Water Treatment Practices			
All Neighbors Reported Boiling Water	Ref	–	–
Has One or More Neighbors who Reported not Boiling Water	0.94	0.77–1.15	0.545
Has No Close Neighbors	0.80	0.61–1.06	0.124
Number of Neighbors	0.99	0.97–1.01	0.339
Had Diarrhea in Past 2 Weeks (vs. Did Not)	1.08	0.91–1.29	0.374

aRR = adjusted relative risk; Ref = reference. Models account for robust standard errors and clustering at the village level.

## DISCUSSION

In this population-based study of women with young children across eight villages in rural Uganda, where there is limited access to clean drinking water, 43% of women misperceived safe water treatment practice as uncommon among peers. Specifically, they incorrectly believed that most women with young children in these villages do not typically treat their household drinking water in any way to make it safer. These misperceptions were present across social categories despite only 17% of women reporting that they do not take action to make their household drinking water safer.

The public health importance of this finding is that women who misperceived local norms about safe water treatment practices (i.e., they incorrectly thought that most did not boil their water) were less likely to treat their households’ drinking water themselves. These two key findings are analogous to phenomena whereby people overestimate peers’ engagement in health risk behaviors, in this context^63^^,^^64^^,^^67^^,^^69^^,^^86^ and elsewhere,^87^^–^^95^ and also analogous to estimates of strong associations that have been observed between perceived norms and personal behavior.^87^

Misperceived norms about water treatment practices might arise due to falsely attributing a behavior witnessed one time (e.g., someone not treating recently collected water before consumption) as typical behavior or by unconsciously extrapolating the behavior of one individual who does not treat their water to the entire group. Similarly, conditional on there being any “everyday discussion” about water treatment practices, if conversation happens in-person or on the news about children sick with diarrhea, then individuals might presume instead that most women (typically the main caregivers) are not treating the household water. Moreover, boiling water may not be a visible behavior, that is, neither regularly discussed nor done in tandem with others outside one’s own household. Not witnessing others engaging in safe water treatment practices (or not paying attention to the act of boiling water, for example) might make these behaviors seem uncommon.

Taken together, the findings from this study highlight an opportunity to implement a social norms approach strategy to increase water treatment practices in places lacking access to clean water. This approach focuses on correcting misperceived norms by disseminating messages about positive behavioral norms that already exist within a community and by making the desired behavior more visibly salient. For example, a verbal or visual norms message could indicate that “most women with young children in your village boil their household drinking water.” The mechanism behind this strategy is that changed perceptions will motivate behavioral change.^43^^,^^44^^,^^96^^,^^97^ This kind of messaging could be conducted alone or complement other local water, sanitation, and hygiene intervention strategies.^98^^,^^99^

Correcting misperceptions among people experiencing false consensus may encourage initiation of safe water treatment practices and then consistent use of such practices. Additionally, this strategy may prevent people who misperceive the norm but treat their drinking water, from relaxing their treatment practices. Highlighting what they personally already do as the true local norm would support their continued engagement in the health-protective behavior. A large body of research on other topics has shown that changing perceived norms drives personal behavior and attitudes.^26^^,^^39^^,^^40^^,^^100^^–^^103^ Finally, correcting misperceptions broadly across the population may increase conversation about safe water treatment practices and reduce silent bystander behavior. For example, this strategy might increase the likelihood that individuals will speak-up when they see unsafe water treatment practices, that they will encourage peers to adopt safer practices, or that they will offer resources (e.g., firewood) to help peers do so. Emphasizing local protective norms could complement other water, sanitation, and hygiene (WASH) strategies to address risk behavior. Further research is needed to assess effectiveness of this type of social norms approach strategy on increasing safe water treatment practices.

There are limitations to these interpretations. First, only one person per household reported their own water treatment practices. If multiple people per household take responsibility for household drinking water, then not collecting reports from everyone in a household about water treatment practices might affect calculation of actual local norms. However, in this setting, mothers are typically responsible for obtaining water and its various household uses. Second, determination of local norms was based on participants’ reports of their own behavior rather than on objective measures. Thus, participants may have reported that they engage in safe water treatment practices when in reality they do not. In studies of chlorine treatment of drinking water, the prevalence of self-reported water treatment is greater than the prevalence of positive chlorine tests, suggesting that social desirability bias may increase the propensity to report water treatment behavior.^104^^,^^105^ Yet almost half of participants in this study reported believing that most others do not use safe water treatment practices. Ostensibly these participants would feel less social pressure in reporting their own risk behavior (i.e., no safe water treatment practice) if they believe that most are not doing so too. Moreover, even if 20% of study participants falsely reported that they treat their water, the majority would still engage in safe water treatment practices. Additionally, although we did not collect information on whether participants were knowledgeable about boiling water as a safe water treatment practice, lack of knowledge is probably not a barrier. A recent study of key informants from this same region suggested that most people in this context treat their water in some way, almost always by boiling the water.^106^ Thus, the local norm in this study would still be to treat household drinking water. Third, the extent and role of misperceived norms may differ in contexts with greater water insecurity and less safe water treatment practice). However, people across contexts underestimate the prevalence of health behavior and overestimate the prevalence of health risk behavior despite contextual differences in the prevalence of a behavior.^89^^,^^107^^,^^108^ Finally, causal interpretation of results is not possible. However, implementing a social norms approach strategy may change misperception and ultimately personal and collective behavior regardless prior causal direction. Several longitudinal and experimental studies in other contexts have found that perceived norms at an earlier time point influence later personal behavior^109^^–^^113^ and that changing norm perceptions leads to changes in behavior and attitude.^45^^,^^48^^,^^51^^,^^52^^,^^114^^–^^120^

Future research is needed to address these questions. For example, future studies on this topic should attempt to collect objective measures of water treatment practices, for example, within a typical week. Additionally, collecting data on personal beliefs and perceived injunctive norms about whether using safe water treatment practices is what households in that context should do would inform the extent to which perceived descriptive norms and perceived injunctive norms may independently or interactively influence behavior. Moreover, in settings where safe water treatment practice may not yet be normative, personal and collective attitudes may support a health behavior even if local behavioral norms do not yet represent that health behavior. At the same time, it is possible that people may also misperceive injunctive norms around local water treatment practices. Another alternative would be to collect data on perceived norms about safe water treatment practices in specific scenarios (e.g., when cooking, or when someone is ill, etc.) and about safe water storage practices. Overall, however, this study provides the foundational evidence for future research to assess the causal effects of changing misperceived norms about water treatment practices on behavior and conversation at the individual and collective levels, and how it could effectively complement other WASH-related strategies.

## CONCLUSION

In this population-based study of water treatment practices where potable water is rare, we found that almost one in five women living with young children do not boil household water or use other safe water treatment practices. Additionally, almost half of women across eight villages incorrectly believed that most other women with young children in their same villages do nothing to treat household drinking water. This misperception was associated with not personally engaging in safe water treatment practices. Implementing strategies to correct these misperceptions may hold promise for increasing both uptake and maintenance of safe water treatment practices among women with young children in water insecure settings.
